# Comparative Analysis of Different Platelet Lysates and Platelet Rich Preparations to Stimulate Tendon Cell Biology: An In Vitro Study

**DOI:** 10.3390/ijms19010212

**Published:** 2018-01-10

**Authors:** Franka Klatte-Schulz, Tanja Schmidt, Melanie Uckert, Sven Scheffler, Ulrich Kalus, Markus Rojewski, Hubert Schrezenmeier, Axel Pruss, Britt Wildemann

**Affiliations:** 1 Julius Wolff Institute, Berlin-Brandenburger Center for Regenerative Therapies, Charité-Universitätsmedizin Berlin, Corporate Member of Freie Universität Berlin, Humboldt-Universität zu Berlin and Berlin Institute of Health, 13353 Berlin, Germany, tanja.schmidt@charite.de (T.S.); melanie.uckert@gmx.de (M.U.); britt.wildemann@charite.de (B.W.); 2 Sporthopaedicum Berlin, 10627 Berlin, Germany; sven.scheffler@gmx.com; 3 Institute of Transfusion Medicine, Tissue Bank, Charité-Universitätsmedizin Berlin, Corporate Member of Freie Universität Berlin, Humboldt-Universität zu Berlin and Berlin Institute of Health, 10117 Berlin, Germany; ulrich.kalus@charite.de (U.K.); axel.pruss@charite.de (A.P.); 4 Institute for Transfusion Medicine, University Hospital Ulm, 89081 Ulm, Germany; markus.rojewski@uni-ulm.de (M.R.); h.schrezenmeier@blutspende.de (H.S.); 5 Institute for Clinical Transfusion Medicine and Immunogenetics Ulm, German Red Cross Blood Donation Service, 89081 Ulm, Germany

**Keywords:** platelet rich plasma, platelet lysate, tenocyte-like cells, tendon healing, cell culture

## Abstract

The poor healing potential of tendons is still a clinical problem, and the use of Platelet Rich Plasma (PRP) was hypothesized to stimulate healing. As the efficacy of PRPs remains unproven, platelet lysate (PL) could be an alternative with its main advantages of storage and characterization before use. Five different blood products were prepared from 16 male donors: human serum, two PRPs (Arthrex, (PRP-ACP); RegenLab (PRP-BCT)), platelet concentrate (apheresis, PC), and PL (freezing-thawing destruction of PC). Additionally, ten commercial allogenic PLs (AlloPL) from pooled donors were tested. The highest concentration of most growth factors was found in AlloPL, whereas the release of growth factors lasted longer in the other products. PRP-ACP, PRP-BCT, and PC significantly increased cell viability of human tenocyte-like cells, whereas PC and AlloPL increased Col1A1 expression and PRP-BCT increased Col3A1 expression. MMP-1, IL-1β, and HGF expression was significantly increased and Scleraxis expression decreased by most blood products. COX1 expression significantly decreased by PC and AlloPL. No clear positive effects on tendon cell biology could be shown, which might partially explain the weak outcome results in clinical practice. Pooled PL seemed to have the most beneficial effects and might be the future in using blood products for tendon tissue regeneration.

## 1. Introduction

Tendon healing is limited due to the poor vascularity and intrinsic healing capacity [1,2,3]. To address this deficit, several therapeutic approaches were investigated in order to optimize tendon healing processes. Over the last decade, different platelet preparations were tested regarding their stimulating effect on musculoskeletal tissue healing [4,5], showing that PRP can enhance human bone, muscle, and tendon cell proliferation as well as the collagen I gene expression and matrix synthesis of tenocytes in vitro [6,7,8]. Furthermore, PRP has been shown to promote tendon healing in vivo using rodent Achilles and Patellar tendon pathology models [9,10,11,12]. Platelet Rich Plasma (PRP) or other blood preparations might offer the possibility to promote tendon healing without having any negative side effects and are low in costs. The idea that PRP can promote tissue healing is based on the high content of growth factors in the alpha granula of the platelets like transforming growth factor β (TGF-β), platelet-derived growth factor (PDGF), insulin-like growth factor 1 (IGF-1), vascular endothelial growth factor (VEGF), fibroblast growth factor (FGF), and others, which are known to trigger or even be involved in angiogenesis and tissue regeneration [13]. However, the efficacy of PRP for clinical applications remain unproven [14,15], but positive effects on pain relief and chronic impairments were reported [16,17,18,19,20,21,22]. Existing studies often lack appropriate controls to validate the findings. The variable outcome data may be due to the high individual differences of each patients PRP composition regarding platelet number and therefore concentration of growth factors. Additionally, methods for preparation, activation of platelets, surgical application and treatment volume are highly variable [23,24,25]. Platelet concentrate (PC) is a standard blood product in transfusion medicine, which is prepared by apheresis. It contains ten times more platelets compared to PRP, nearly no other blood cells and the platelet content is nearly independent from the donors age and sex. Therefore, we choose PC as our source material to prepare platelet lysate (PL), a cell free supernatant rich in growth factors, which are released from the platelets after freeze-thawing disruption of the PC [26]. PL is already clinically used for the treatment of different regenerative pathologies of wounds and eye ulcers [27,28,29,30]. Additionally, in vitro studies showed the potential of PL to enhance wound healing processes [31,32]. A further advantage of PL compared to conventional PRPs is that it can be stored frozen and therefore used for consecutive applications. Furthermore, it can be analyzed for growth factor content and other characteristics before its use to standardize the product. As PC is supposed for allogenic use with an already established safety/testing system, an allogenic use for PL might also be conceivable. Although PRP and modifications like PL are intensively investigated during the last years, existing studies mostly focus on PL as a humane based cell culture supplement for preclinical cell propagation [33,34,35]. Furthermore, studies mainly investigated the impact of PL on humane MSC’s, chondrocytes or corneal endothelium cells [36,37,38,39]. To our knowledge, only one study exists, which investigated the impact of PL on tenocytes in vitro [40]. Furthermore, there is a lack of studies that compared PLs to standard PRPs regarding their growth factor content as well as their stimulatory potential on human tenocytes of the rotator cuff.

Therefore, it was the aim of the present study to characterize five different platelet-based blood products including two PLs, two PRPs, and PC and compare their capacity to affect cell viability and gene expression of several markers related to tendon extracellular matrix, modeling and remodeling, inflammation, and pain. The hypothesis was that PC and both PLs would have advantageous effects compared to standard PRP preparation due to their increased platelet content and possibly increased growth factor content.

## 2. Results

Blood from 16 donors was taken, and all four different blood products were produced from the blood of each donor to allow the comparison.

### 2.1. Characterization of Blood Products

**Figure 1:**
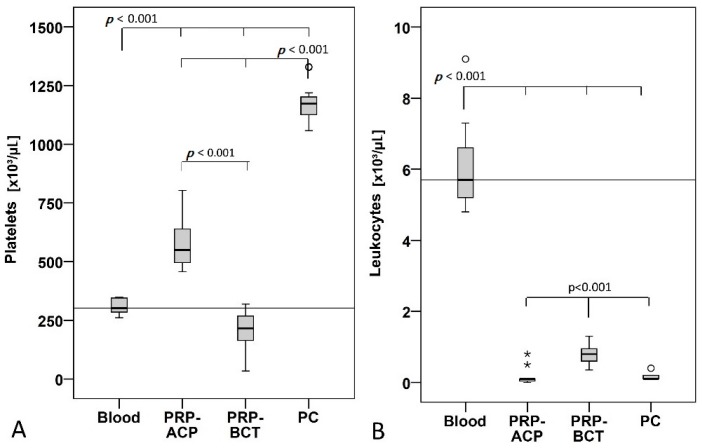
Platelet (**A**) and leukocyte (**B**) concentration in Arthrex, (PRP-ACP), RegenLab (PRP-BCT), and platelet concentrate (PC) compared to whole blood. (**A**) Platelet concentration was significantly higher in PRP-ACP and PC group and lower in the PRP-BCT group. (**B**) Leukocyte concentration was significantly reduced in all groups. °^,^* indicate outliers, *n* = 16 individual donors, all blood product were produced from each donor.

The concentration of platelets and leukocytes was quantified in the whole blood and the blood products PRP-ACP, PRP-BCT, and PC and from each individual donor. The strongest enrichment of platelets was found in the PC (3.8 fold higher than blood) followed by PRP-ACP (1.9 fold higher than blood). Surprisingly, PRP-BCT had a reduced platelet count (0.7 fold lower compared to blood). Significant differences between the groups are shown in Figure 1A. Leukocytes were significantly reduced in all blood products compared to the whole blood. PRP-BCT showed a significantly increased leukocyte content compared to PRP-ACP and PC (Figure 1B). PCs used to produce pooled AlloPL contained between 500–2045 × 10^3^ platelets/µL according to manufacturer information (data sheet) and were leukocyte depleted (<5 leukocytes/µL).

**Figure 2:**
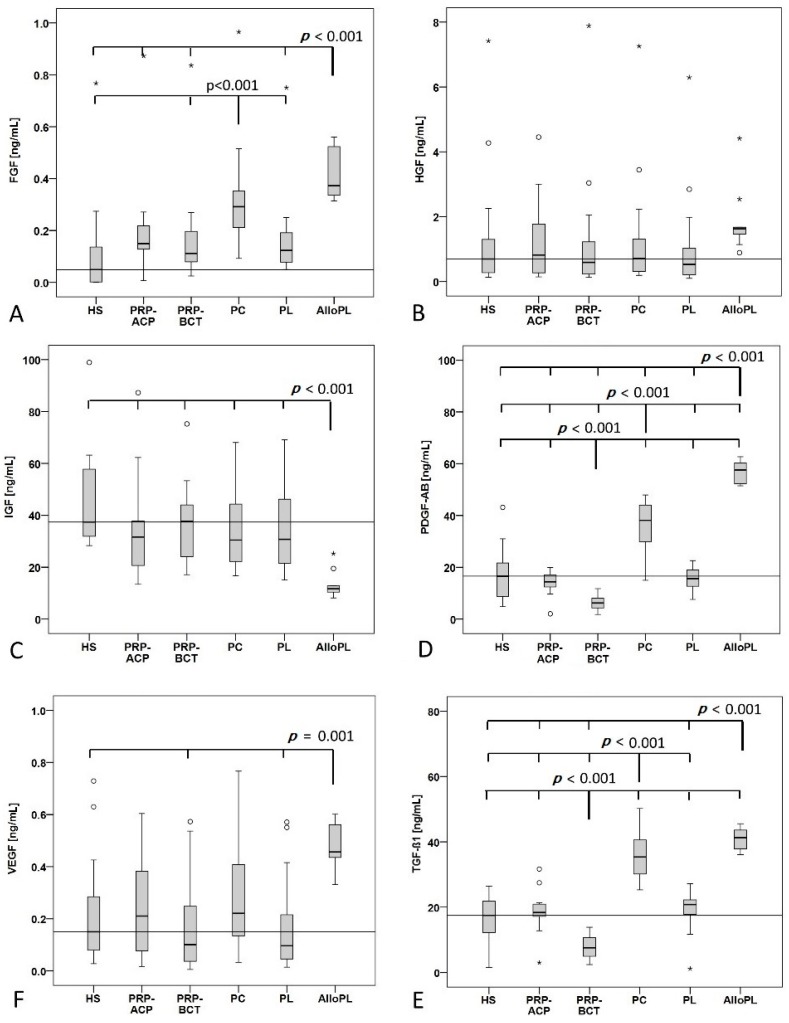
Growth factor quantification in the blood products PRP-ACP, platelet rich plasma (PRP), RegenKit-Blood Cell Therapie (BCT), PC, platelet lysat (PL), Allogenic platelet lysate (AlloPL), and human serum (HS) control measured by ELISA. (**A**) Basic fibroblast growth factor (bFGF) concentration was higher in PC compared to HS, PRP-BCT, and PL as well as in AlloPL compared to HS, both PRPs, and PL. (**B**) Hepatocyte growth factor (HGF) concentration was not significantly changed. (**C**) Insulin-like growth factor 1 (IGF-1) concentration was decreased in the AlloPL group. (**D**) Platelet-derived growth factor (PDGF-AB) and (**E**) transforming growth factor β (TGF-β1) concentration was lower in the PRP-BCT group and higher in the PC and AlloPL group compared to all other groups and for TGF-β1 concentration except for PC and AlloPL. (**F**) Vascular endothelial growth factor (VEGF) concentration was increased in the AlloPL group compared to HS, PRP-BCT, and PL. °^,^* indicate outliers, *n* = 16, except for AlloPL *n* = 10.

Growth factor quantification was performed for all blood products and human serum (HS) as control (Figure 2). bFGF concentration was significantly increased in the PC group compared to HS control, PRP-BCT and PL group. Additionally, AlloPL showed a significantly higher bFGF concentration compared to all other groups except PC. HGF concentration did not differ between the blood products and the HS, while IGF-1 concentration was significantly decreased in the AlloPL group compared to all other groups. PDGF-AB and TGF-β1 showed a similar pattern with a decreased concentration in the PRP-BCT group compared to all other groups and an increased concentration in the PC and AlloPL group compared to all other groups. The TGF-β1 concentration did not differ between PC and AlloPL. The concentration of VEGF was significantly higher in the AlloPL group compared to HS, PRP-BCT and PL. The outliners in bFGF, HGF, and VEGF concentration came from the same two blood donors.

Negative moderate correlation was found for the platelet concentration with the leukocyte concentration (r_s_: −0.608). Additionally, a strong positive correlation of platelet concentration was seen with PDGF-AB and TGF-β1 (r_s_: 0.850 and r_s_: 0.837).

**Figure 3:**
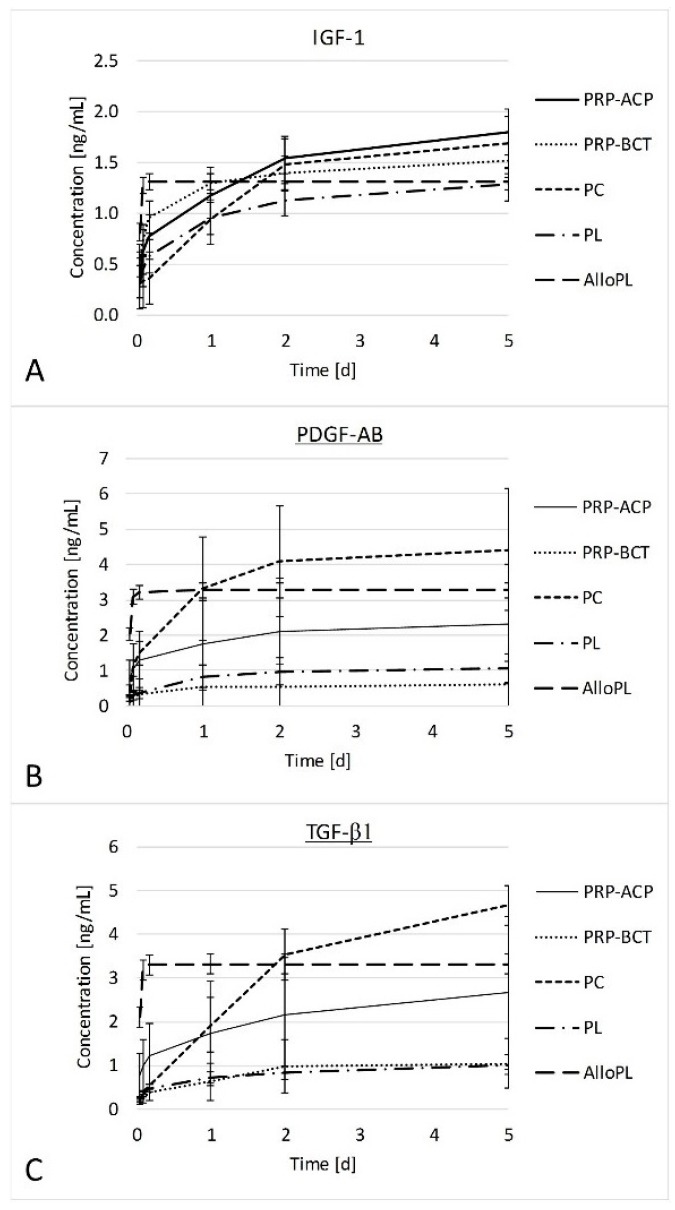
Cumulative growth factor release from blood products into the medium measured after 1, 4, 24, 48, and 120 h by ELISA. IGF-1 (**A**), PDGF-AB (**B**), and TGF-β1 (**C**) were release only over 4 h by AlloPL but constantly over 2–5 days by the other blood products. The release experiments were performed exemplarily for *n* = 4 donors.

In the release experiment, the blood product was placed into a cell culture well and the growth factor concentrations secreted into the medium over a five-day period were evaluated (Figure 3). bFGF, HGF, and VEGF concentrations were below the detection limit of the assays in most cases. Only in the AlloPL group mean amounts of 21.7 ± 2.1 pg/mL bFGF and 56.2 ± 64.1 pg/mL HGF were found after 1 h. IGF-1, PDGF-AB and TGF-β1 were released from PRP-ACP, PRP-BCT, PC and PL for at least 2 days and in some cases also over the entire period of five days. From AlloPL the growth factors were released only at the early time points until 4 h. PRP-ACP reached the highest final concentration of IGF-1 followed by PC and PRP-BCT. PDGF-AB and TGF-β1 showed a comparable release pattern. PC reached the highest final concentrations of PDGF-AB and TGF-β1 followed by AlloPL, PRP-ACP, and finally PL and PRP-BCT.

### 2.2. Cell Stimulation

**Figure 4:**
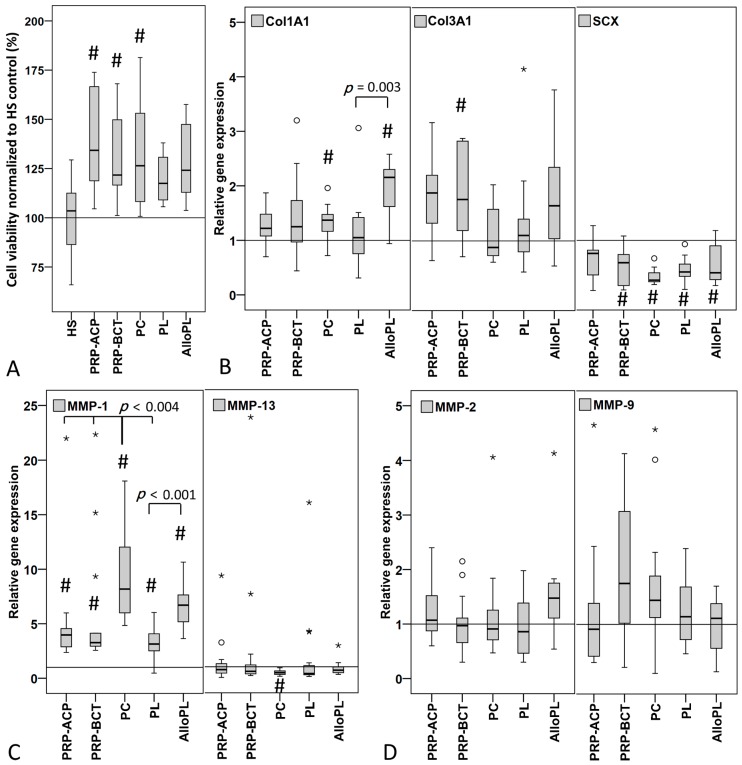
Cell viability and relative gene expression in human tenocyte-like cells (hTLCs) stimulated with blood products compared to HS control (line) measured by qPCR using Δ*C*_t_ method with efficiency correction normalized to 18S rRNA. (**A**) Cell viability was significantly increased by both PRPs and PC compared to HS control. (**B**) Col1A1 expression was significantly increased by PC and AlloPL group compared to HS control and in AlloPL compared to PL. Col3A1 expression was significantly increased by PRP-BCT and scleraxis (SCX) expression decreased in all groups except PRP-ACP compared to HS control. (**C**) MMP-1 expression significantly increased by all blood products compared to HS control with significantly highest expression in the PC group and MMP-13 decreased by PC stimulation. (**D**) MMP-2 and MMP-9 expression did not change. # marks significant differences between the HS control and the blood products and the spanning line between the individual groups. °^,^* indicate outliers. PRP-ACP: *n* = 11, PRP-BCT: *n* = 12, PC: *n* = 15, PL: *n* = 14, AlloPL: *n* = 10. N-numbers varied due to clotting events in the well, which leads to the loss of cells.

Cell viability measured by Alamar Blue Assay of the human tenocyte like cells (hTLCs) increased significantly when stimulated for five days with PRP-ACP, PRP-BCT, and PC compared to the control stimulation with HS (Figure 4A). No significant differences could be observed for the comparison between the individual blood products. Cell viability correlated in a negatively moderate fashion with the leukocyte content (*r*_s_ = −0.517, *p* ≤ 0.001).

The expression of the extracellular matrix marker Col1A1 was significantly increased in the hTLCs stimulated with PC and AlloPL (Figure 4B). Additionally, the AlloPL-stimulated cells showed an increased Col1A1 expression compared to PL stimulated cells. Col3A1 expression was significantly increased after stimulation with PRP-BCT (Figure 4B). The expression of the tendon-related transcription factor scleraxis (SCX) was significantly decreased in all groups except for PRP-ACP (Figure 4B). In the group of the matrix degrading enzymes, the expression of the collagenase MMP-1 was significantly increased in hTLCs by all blood products compared to the HS control, while additionally the PC stimulated cells showed an increased expression compared to both PRPs and PL. AlloPL stimulation significantly increased MMP-1 expression compared to PL. The expression of the collagenase MMP-13 significantly decreased after PC stimulation in the hTLCs (Figure 4C). No alterations of the expression of the gelatinases MMP-2 and MMP-9 could be observed after stimulation (Figure 4D).

**Figure 5:**
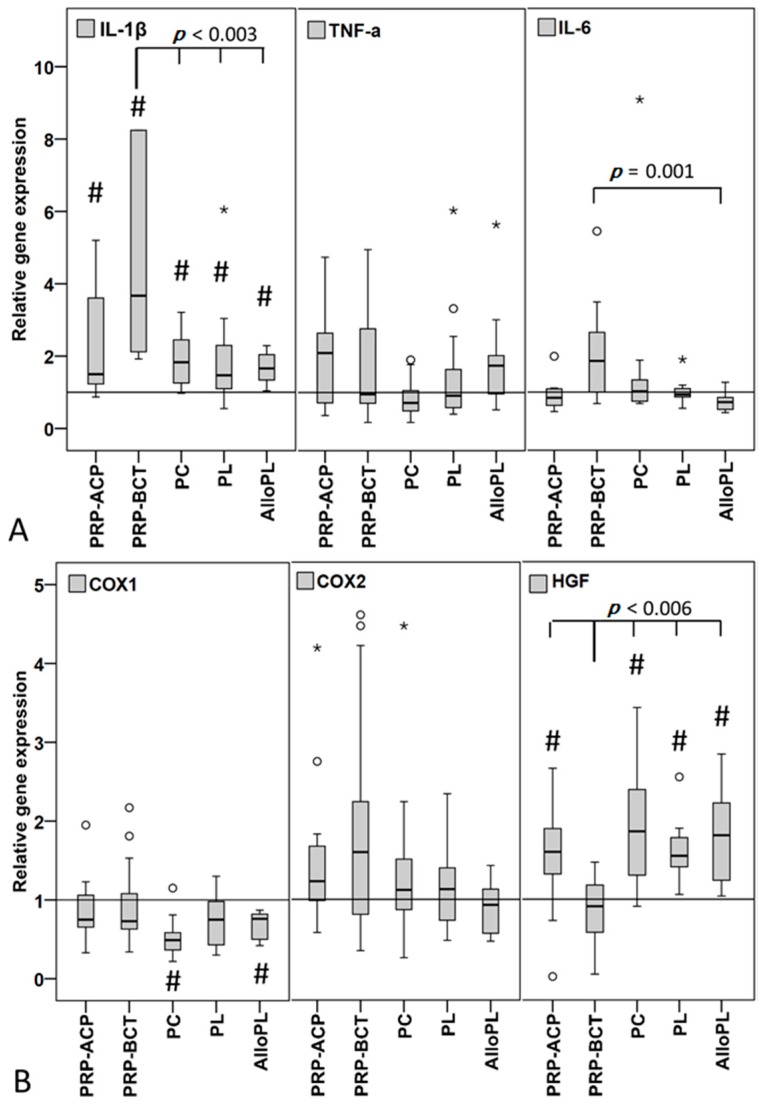
Relative gene expression in hTLCs stimulated with blood products compared to HS control (line) measured by qPCR using Δ*C*_t_ method with efficiency correction normalized to 18S rRNA. (**A**) IL-1β expression was significantly increased by all blood products with significantly highest expression in PRP-BCT group (higher outliners were cut off). TNF-α was not altered in all groups. IL-6 was decreased in the AlloPL group compared to PRP-BCT. (**B**) COX1 expression was significantly decreased in PC and AlloPL group and COX2 expression did not change. HGF expression was significantly increased by all products except PRP-BCT. # marks significant differences between the HS control and the blood products and the spanning line marks significant differences between the individual groups. °^,^* indicate outliers. PRP-ACP: *n* = 11, PRP-BCT: *n* = 12, PC: *n* = 15, PL: *n* = 14, AlloPL: *n* = 10. N-numbers varied due to clotting events in the well, which leads to the loss of cells.

The stimulation of hTLCs with all blood products resulted in a significantly increased expression of the pro-inflammatory cytokine IL-1β. The strongest but also highly variable increase was observed after PRP-BCT incubation (Min-Max relative gene expression: 0.87–362.45), which was significantly different compared to the PC, PL and AlloPL group (Figure 5A). The TNF-α expression was not affected by the stimulation with the blood products and IL-6 expression was decreased by AlloPL application compared to the PRP-BCT group. The expression of the anti-inflammatory cytokine IL-10 revealed no or negligible amounts of RNA in the analyzed hTLCs. The pain related factors COX1, COX2 and HGF were regulated in a varying manner (Figure 5B). The stimulation of the hTLCs with the blood products PC and AlloPL significantly decreased the COX1 expression, while COX2 expression was not affected by treatment with the blood products. HGF expression was significantly increased compared to HS control by all blood products except for PRP-BCT. PRP-BCT stimulation additionally showed a decreased HGF expression in the hTLCs compared to all other blood products.

A positive moderate correlation was found for MMP-1 and HGF expression with the platelet concentration (MMP-1: *r*_s_ = 0.513, *p* ≤ 0.001; HGF: *r*_s_ = 0.648, *p* ≤ 0.001), and a moderate negative correlation was observed with the leukocyte content of the blood products (MMP-1: *r*_s_ = −0.626, *p* ≤ 0.001; HGF: *r*_s_ = −0.578, *p* ≤ 0.001). For the HGF expression, a moderate correlation with the TGF-β1 concentration could be observed (*r*_s_ = 0.539, *p* ≤ 0.001).

## 3. Discussion

The aim of the present study was to compare the effect of five different platelet-based blood products on tenocytes of the human rotator cuff to better understand their possible effect in clinical use. Next to the commercially available PRPs PRP-ACP and PRP-BCT and a platelet concentrate (PC) obtained by apheresis, the focus of the present study was to analyze two platelet lysates, one conducted from PC (PL) and one commercially available pooled PL from different donors (AlloPL). The two PLs have application advantages such as characterization before use and storage for repeated use.

Our hypothesis, that products with a higher platelet content like PC and its preparation PL as well as the pooled AlloPL would have advantageous effects compared to standard PRP preparations due to their possibly increased growth factor content, has to be partly rejected. Although PC and AlloPL showed an increased content of the growth factors TGF-β1, VEGF, PDGF-AB, and bFGF, this was not accompanied by highly increased stimulation of tenocytes viability and gene expression of extracellular matrix proteins. However, the stronger effects on HGF expression as well as the downregulation of COX1 expression seen after stimulation with PC and AlloPL suggest stronger effects on pain relief and inflammation compared to standard PRP’s.

Surprisingly, PL showed significantly different results, especially compared to PC and AlloPL, although the initial platelet concentration was comparable in the three products. Whereas AlloPL showed the highest growth factor concentrations except for IGF-1 within all blood products, PL showed relatively low concentrations. The preparation method of both PLs might be the reason for the different growth factor contents. First, AlloPL was prepared from PCs, which were stored up to six days before further preparation. During that storage time, further platelets might be activated and produced growth factors and therefore increased growth factor concentration in the AlloPL after platelet lysis. The positive influence of longer storage of PLs on the concentration of most growth factors and the decrease in IGF-1 concentration was shown previously [41]. Furthermore, PL was produced by a freezing step at −80 °C compared to −30 °C for the AlloPL, which might have destroyed some growth factors and reduced their impact. The −80 °C freezing temperature was chosen in establishing experiments to be the best temperature to reduce clotting events in the well [42].

A strength of the study is the preparation of all four blood products and HS (except of AlloPL) from the same 16 donors. This allows a direct comparison of the results and reduces the donor dependent variations. However, variations in the growth factor content due to the donors are quite obvious: distinct variations and outliners as shown in Figure 2. Analyzing the in vitro release of the growth factors, AlloPL showed a burst release of growth factors into the medium until 4 h, whereas a more continuous release was observed for the other blood products. This might indicate that in AlloPL all platelets were destroyed and therefore released their growth factors, whereas in PL the freezing process might have been not sufficient to destroy all platelets to release their growth factors. However, the different release kinetics could also be caused by different clotting intensities of the blood products in the transwell insert, whereas a denser clot as seen for PRP-ACP, PRP-BCT, PL, and PC leads to a more continuous release compared to AlloPL. This confirms other studies demonstrating the influence of PRP clot characteristics on the growth factor release pattern [43,44]. A comparable release pattern was observed by other authors investigating the release from PRP clots [43], whereas the release pattern from liquid PRPs is more comparable to the present AlloPL [45]. It was speculated that a burst release of growth factors decreases the therapeutic efficacy of PRPs [43]. Unfortunately, this speculation cannot be proved by the in vitro study. The half-life of the growth factors in vitro and in vivo is not comparable and it is expected that it is shorter in vivo. Therefore, the initial release from AlloPL is sufficient to stimulate the cells, as demonstrated within the present study.

As expected platelet content was highest in PC followed by ACP and BCT. Surprisingly, the PRP-BCT was not able to reach a platelet count comparable to the whole blood (concentration factor: 0.7). A handling mistake can be excluded, as we were instructed by the company at the first PRP-BCT preparations. In PRP-ACP, increased platelet concentrations were found (concentration factor: 1.8), which was comparable to other studies [7,46]. Another study confirmed variations in the platelet content between PRPs from Arthrex and Regenlab with, in contrast, a higher concentration factor in the PRP from Regenlab compared to Arthrex [47]. The varying findings might be a result of variations in the preparation method, such as anticoagulant as used for the Arthrex PRP preparation in this study, whereas our PRP-ACP was produced without anticoagulate, as it is performed in clinical practice in our hospital.

The strong variations in platelet and growth factor concentrations between the blood products do only weakly affect the cell viability or gene expression of the hTLCs. The high growth factor concentrations in the PC and AlloPL did not result in highly increased cell viability, which was increased strongest by both PRPs and PC. This underlines that a high platelet or growth factor concentration, especially for those growth factors regulating cell growth, did not result in highly increased cell viability and can have stagnating or inhibitory effects, as also reported by other authors [48,49,50,51,52]. A comparable positive effect of PRP-ACP on the cell proliferation of tenocytes was previously shown [7].

Also, the expression data weakly mirror the strong variation in platelet and growth factor concentration. Only the Col1A1 and MMP-1 expression was increased the most by PC and AlloPL. Other studies also showed that the regulation of Col1A1 expression after treatment of tenocytes with platelet based blood products seems to be highly dependent on the concentration and activation of the product and the used negative control and was found to be increased, not regulated or decreased [8,50,51,52]. Col3A1 is associated with the reduction of biomechanical properties of the tendon [53] and the increase after stimulation with PRP-BCT might negatively affect the tendon healing outcome. Comparable to PRP effects on Col1A1 expression, varying results were reported for Col3A1 with increased and decreased Col3A1 expression after stimulation of tenocytes with different platelet-based blood products [8,51,52]. The expression of the tendon related transcription factor SCX was in the present study decreased by all blood products except for PRP-ACP. SCX is important for tendon formation and development [54], and it was shown that SCX transduction of human MSCs led to their reprogramming into tendon progenitors [55]. A decrease might therefore lead to a dedifferentiation of the hTLCs and might in vivo result in the formation of a less organized tendon structure as demonstrated for Scx−/− mutant mice [56]. A further study stimulating tenocytes with blood products found a comparable decrease in SCX expression [8], whereas others found no regulations or an increased SCX expression [48,50,51]. The higher platelet concentrations and the comparison to 1% or 2% FCS control instead of a 10% HS control might account for the contrary findings, because the higher serum control itself leads to a stronger stimulating effect. The expression of the MMPs differed in the present study. The collagenase MMP-1 was strongly increased by all groups, whereas MMP-13 and the gelatinases showed no changes or a decreased expression. Comparable results were previously found for MMP-1 and MMP-13 expression after stimulation of tenocytes with blood products [52], whereas in contrast, an increased MMP-2 and MMP-9 enzyme activity was found [48]. In the present study, only expression and no enzyme activities were tested, which impede the direct comparison of the results. MMPs are important for the modeling and remodeling of the extracellular matrix of tendons and the balance between MMPs and their natural inhibitors; the Tissue Inhibitors of Metalloproteases (TIMPs) are important to maintain tendon homeostasis [57]. The strong increase in MMP-1 expression might lead to an imbalanced MMP/TIMP ratio and might therefore negatively affect the tendon healing process. 

The leukocyte concentration in the present study was higher in the PRP-BCT group compared to the PRP-ACP and PC group. This confirms another study showing that PRP-ACP has a lower amount of leukocytes compared to PRP-BCT. However, the authors found a leukocyte concentration factor of 1.52 in the PRP-BCT compared to whole blood, whereas we observed for both PRPs a leukocyte-reduction compared to whole blood (concentration factor PRP-ACP: 0.026; PRP-BCT: 0.13) [47]. Even this underlines that, despite the compliance with the manufacturer’s instructions, the PRP composition highly depends on the individual preparation method. The role of leukocytes in PRP is still under debate. One study claims an important antimicrobial role of leukocytes [58], while another study refers to a negative relationship between leukocytes and healing [59]. With the analysis of the expression of inflammatory cytokines (IL-1β, TNF-α, IL-6, IL-10) in the stimulated hTLCs, we aimed to investigate the effect of the blood products on inflammatory processes. IL-1β expression was increased by all blood products, but strongest by PRP-BCT, which also showed the highest leukocyte concentration. Additionally, a correlation between leukocytes and the IL-1β concentration could be observed. We did not analyze the concentration of cytokines in the blood products itself, but the presence of IL-6 and TNF-α in blood products was shown previously [50]. Additionally, the IL-1β concentration was linked to neutrophils and monocytes in PRPs [46]. As shown in a cell culture study, the stimulation of tenocytes with TNF-α led to an up-regulation of IL-1β expression [60]. The present results might indicate that leukocytes and/or cytokines in the blood products induce pro-inflammatory processes in the cell. It stays questionable if this is positive or negative for the tendon healing process and the later outcome.

The published PRP studies demonstrate the complexity of comparison between each other, due to different preparation methods, platelet/growth factor concentration, activation and stimulation time and the used negative control (1% FCS, 2% FCS, 10% FCS, and 10% human serum). Most cell culture studies claim positive results of different PRPs but so far the clinical observations are restrained. Most authors reported on no positive effects of PRPs on the functional clinical outcome after tendon repair [61,62,63,64]. However, a positive effect on pain reduction was observed [21,65]. Therefore, also the expression of the pain associated factors COX1, COX2, and HGF were analyzed. Zhang et al. claimed an anti-inflammatory effect of PRP due to the presence of HGF. And this in turn was hypothesized to result in pain reduction by down regulation of COX1 and -2, prostaglandin (PGE), and PGE synthase [66]. The anti-inflammatory effect of PRP cannot be confirmed by the present results, due to the overall increased expression of the pro-inflammatory cytokine IL-1β. However, the COX1 expression was significantly decreased by PC and AlloPL in the hTLCs. Additionally, the HGF expression was induced by application of all blood products except PRP-BCT. This might result in pain reduction when it comes to PRP treatment in clinical practice.

As a limitation of the study, it has to be mentioned that only hTLCs of old donors (67–72 years) were used to study the effect of the blood products. As we could previously show that the biological characteristics and the stimulation potential after BMP-2/-7 application of hTLCs is decreased with age [67], the use of hTLCs from younger donors could have demonstrated different results. However, old donors were chosen as they represent the typical patient cohort for degenerative rotator cuff ruptures, where the application of blood products might be a treatment option. Another limitation is the missing evaluation of the sex factor in the current study, as we only treated male cells with male blood products. Xiong et al. recently compared PRPs from male and female donors and found significant differences regarding growth factor content between male and female PRPs [68]. To reduce variabilities, we decided to use only male cells and blood products. However, possible sex based differences are an interesting topic and should be investigated in future studies. We are aware that conclusions drawn from the present results are speculative regarding any in vivo effect e.g., with regards to inflammation, but might be very useful to understand the role of different blood products on the cellular level.

## 4. Materials and Methods

### 4.1. Preparation of Human Blood Products

All blood products except for the allogenic platelet lysate (AlloPL) were obtained from 16 healthy male donors with a mean age of 42 years (range 30–50 years). The blood was taken in the Institute of Transfusion Medicine (Berlin, Germany) during routinely blood donation. All donors gave their written informed consent for the production of blood products and usage for the present study (EA1/038/14). The platelet concentration was automatically quantified with the ABX penta XL 80 (Horiba medical, Grabels, France) system and the leukocyte concentration was manually quantified using a Nageotte counting device. 

### 4.2. Platelet Concentrate (PC)/Plasma Lysate (PL)/Allo-PL Preparation

Platelet concentrate (PC) was produced in the Institute of Transfusion Medicine (Berlin, Germany) using a Trima Accel^®^ (TERUMO BCT, Inc., Lakewood, CO, USA) automated blood collection system with a leukocyte reduction system chamber. The PC was used freshly after preparation.

Platelet lysate (PL) was produced from PC by a freezing and thawing step. A total of 5 mL PC was frozen a −80 °C for 30 min to destroy the platelets and release the growth factors. After thawing at 37 °C in the water bath, the lysate was centrifuged at 1600× *g* for 10 min to separate the cell debris. The supernatant was used for cell stimulation.

Allogenic platelet lysate (AlloPL) was obtained from the Institute for Clinical Transfusion Medicine and Immunogenetics, Ulm, Germany. The AlloPL was prepared as described by Fekete et al. from a platelet pool from up to 100 donors and stored at −30 °C [69]. The frozen AlloPL was shipped to our laboratory. Before use the AlloPL was thawed at 37 °C in the water bath and centrifuged at 1800× *g* for 10 min. A total of 10 different AlloPLs were included in the study. The lower number of AlloPL resulted from the expectation of lower variation in the pooled blood product compared to the blood products obtained from individual donors.

### 4.3. Standard PRP Preparations and Human Serum (HS) Control

Platelet rich plasma (PRP) was produced using two different commercially available devices. Autologous conditioned plasma (ACP double syringe system, Arthrex, Germany) was used to produce PRP-ACP according to the manufacturer´s instructions. A total of 10 mL blood was taken into the double syringe without anticoagulate and centrifuged at 400× *g* for 5 min in the Rotofix 32A centrifuge (Hettich, Germany). The upper separated PRP-ACP was subtracted with the inner syringe and used for hTLC stimulation. PRP-BCT was produced with the RegenKit-Blood Cell Therapie (BCT, Regenlab, Le Mont-sur-Lausanne, Switzerland) according to the manufacturer’s instructions. Therefore, 8 mL of blood were directly collected into the RegenKit-BCT tubes containing sodium citrate as anticoagulate and centrifuged at 1500× *g* for 5 min. Afterwards the tubes were slowly pivoted 15 times and the supernatant (PRP-BCT) used for cell stimulation.

Human serum (HS) served as negative control and was produced using a commercially available serum tube. The blood was left to clot for 30 min at room temperature before centrifuged for 10 min at 1500× *g*.

### 4.4. Growth Factor Quantification

For further characterization of the blood products, the concentration of the growth factors basic fibroblast growth factor (bFGF), platelet derived growth factor (PDGF-AB), transforming growth factor β (TGF-β1), hepatocyte growth factor (HGF) (ELISA recognizes VEGF_121_, VEGF_165_, VEGF_165b_), and insulin-like growth factor 1 (IGF-1) were determined using commercially available sandwich ELISAs (DuoSet ELISA, R&D Systems, Wiesbaden, Germany). The frozen blood products were thawed and centrifuged for 5 min at 1600× *g*. The supernatant was used for ELISA. ELISAs were performed according to the manufacturer’s instructions. For the optimal release of the growth factors IGF-1 and TGF-β1, the blood products had to be activated using HCL according to the manufacturer instructions and were afterward neutralized using Tris-Base or NaOH/Hepes, respectively.

### 4.5. Growth Factor Release from Blood Products

The release of growth factors from the blood products over 120 h was analyzed in vitro (*n* = 4 donors). Therefore, the experimental setup was done as described for stimulation experiments, but without cells. After 1 h, 2 h, 4 h, 24 h, 48 h, and 120 h the entire medium was collected and replaced by fresh experimental medium (medium + HS). The elution samples were stored at −20 °C until quantified by sandwich ELISA for the growth factors FGF, HGF, IGF-1, PDGF-AB, TGF-β1, and VEGF. Experimental medium only served as control. 

### 4.6. Human Tenocyte-Like Cells

Human tenocyte-like cells (hTLCs) were obtained from torn supraspinatus tendons from four male patients with a mean age of 69.5 years (67–72 years) undergoing arthroscopic or open surgery for rotator cuff repair of chronic ruptures. All samples were collected according to a standardized protocol and were grasped 3 to 5 mm from the torn proximal tendon edge. Prior to biopsy, all patients gave their written informed consent and the study was approved by the local authorities (EA/060/09). After collagenase digestion, hTLCs were grown in DMEM/HAM’s F12 (1:1) medium with 10% Fetal calf serum (FCS) and 1% Penicillin/Streptomycin (P/S). Cells were trypsinized, pooled, and frozen until used for stimulation experiments. The hTLCs were harvested according to a previously established protocol [70], which proved the isolation of cells with tenocyte-like properties, such as expression of tendon related genes and a distinct expression pattern compared to other cells of the musculoskeletal system.

### 4.7. Cell Stimulation

**Figure 6:**
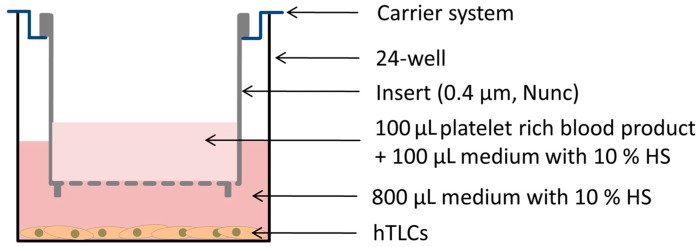
Experimental setup for stimulation of hTLCs with platelet-rich blood products.

A total of 1 × 10^4^ vital cells per well of the pooled hTLCs in passage 2 were seeded into a 24-well plate and incubated for two days in normal growth medium (DMEM/HAMs F-12, 10% FCS, 1% P/S). At day 0 of stimulation, an Alamar Blue test (Biozol, Germany) was performed according to the manufacturer’s instructions to analyze the metabolic activity of the cells and is according to the manual termed as “cell viability” in the text. Afterwards, 800 µL of experimental medium (DMEM/HAMs F-12, 10% HS, 1% P/S) was pipetted into each well. The hTLCs of the negative control received 1 mL of experimental medium. A total of 100 µL of the particular blood products (PC, PL; PRP-ACP, PRP-BCT, AlloPL) and 100 µL experimental medium were mixed and incubated in polycarbonate transwells with 0.4 µm pore size (Nunc, Germany) at 37 °C for 3 h to enable a clotting. The transwells were hung into a carrier plate and applied to the hTLCs in experimental medium, resulting in a concentration of 10% (*v*/*v*) blood products (Figure 6). All stimulations were performed in triplicates. After incubation of the cells with the blood products for five days at 37 °C the inserts were carefully removed from the cells and cell viability was tested again. Afterward, the RNA was isolated with the NucleoSpin RNA Kit (Macherey-Nagel, Germany).

### 4.8. Gene Expression Analysis

RNA quantity and purity was analyzed with the Nanodrop ND1000 system. A total of 100 ng RNA were transcribed into cDNA with the qScript cDNA Supermix (Quanta Biosciences, Beverly, MA, USA). For gene expression analysis, 1.25 ng of cDNA was used as PCR template. Quantitative Real-Time PCR (qPCR) was performed with the SyBr Green Mastermix (Quanta biosciences) according to the manufacturer’s instructions using the Light Cycler 480 System (Roche, Mannheim, Germany). All primer sequences were designed using Primer 3 software (Freeware; Available online: http://frodo.wi.mit.edu/primer3), and were produced by Tib Molbiol, Berlin, Germany (Primer sequences see Table 1). All primers were tested for amplification efficiency and the Δ*C*_t_ method with efficiency correction was used to calculate the relative gene expression to the reference gene 18S rRNA.

### 4.9. Statistics

Statistical analysis was performed using SPSS 20 (IBM, Armonk, NY, USA). Data are presented as boxplots with median and 25% and 75% percentiles and the outliners marked as stars or circles. The Kruskal–Wallis Test was used to determine significant differences between all groups and the Mann–Whitney *U* test was used to evaluate differences between two groups followed by Bonferroni-Holm-Correction to adjust the *p*-value. Statistical significances are given as exact significances with # marking differences to the HS control and a spanning line indicating differences between the blood product groups. Additionally, a Spearmans Rho correlation (*r*_s_) analysis was performed, and correlations above *r*_s_ = 0.5 were considered.

## 5. Conclusions

Taken together, the overall results demonstrate no clear positive stimulatory effect of the different blood products on tendon cell biology due to the increase in pro-inflammatory cytokine IL-1β and matrix degrading enzyme MMP-1 and a decrease in the tendon marker SCX. This might partially be a reason for the weak outcome in clinical practice. AlloPL seems to have the best effect by strongly increasing Col1A1 expression and the pain antagonist HGF and decreasing the pain marker COX1. AlloPL is a pooled lysate of different donors, which might account for the positive findings. Therefore, pooled and well-characterized platelet lysates could be the future for tendon tissue regeneration.

## Figures and Tables

**Table 1:** Primer sequences.

Gene	Accession No.	Primer Sequence	Size (bp)
18S RNA	NM_022551	Forward: 5′ CGGAAAATAGCCTTTGCCATC 3′	107
		Reverse: 5′ AGTTCTCCCGCCCTCTTGGT 3′	
Col1A1	NM_000088.3	Forward: 5′ TGA CCT CAA GAT GTG CCA CT 3′	197
		Reverse: 5′ ACC AGA CAT GCC TCT TGT CC 3′	
Col3A1	NM_000090.3	Forward: 5′ GCT GGC ATC AAA GGA CAT CG 3′	199
		Reverse: 5′ TGT TAC CTC GAG GCC CTG GT 3′	
IL-1β	NM_000576	Forward: 5′ TCC AGG AGA ATG ACC TGA GC 3′	111
		Reverse: 5′ GTG ATC GTA CAG GTG CAT CG 3′	
IL-6	NM_000600	Forward: 5′ TGA GGA GAC TTG CCT GGT GA 3′	188
		Reverse: 5′ TTG GGT CAG GGG TGG TTA TT 3′	
IL-10	NM_000572	Forward: 5′ TGA GAA CAG CTG CAC CCA CT 3′	164
		Reverse: 5′ GGC AAC CCA GGT AAC CCT TA 3′	
TNF-α	NM_000594	Forward: 5′ AGC CCA TGT TGT AGC AAA CC 3′	133
		Reverse: 5′ GAG GTA CAG GCC CTC TGA TG 3′	
COX1	NM_001271368	Forward: 5′ CGT GTG TGT GAC CTG CTG AA 3′	193
		Reverse: 5′ TGC GGT ATT GGA ACT GGA CA 3′	
COX2	NM_000963	Forward: 5′ TAG AGC CCT TCC TCC TGT GC 3′	129
		Reverse: 5′ TGG GGA TCA GGG ATG AAC TT3′	
HGF	NM_000601	Forward: 5′ CGC TGG GAG TAC TGT GCA AT 3′	116
		Reverse: 5′ GCC CCT GTA GCC TTC TCC TT 3′	
MMP-1	NM_002421.3	Forward: 5′ CAC GCC AGA TTT GCC AAG AG 3′	148
		Reverse: 5′ GTC CCG ATG ATC TCC CCT GA 3′	
MMP-2	NM_004530	Forward: 5′ TGG ATG ATG CCT TTG CTC GT 3′	156
		Reverse: 5′ CCA GGA GTC CGT CCT TAC CG 3′	
MMP-9	NM_004994.2	Forward: 5′ GGG ACG CAG ACA TCG TCA TC3′	150
		Reverse: 5′ GGG ACC ACA ACT CGT CAT CG 3′	
MMP-13	NM_002427.3	Forward: 5′ CCT TCC CAG TGG TGG TGA TG 3′	144
		Reverse: 5′ CGG AGC CTC TCA GTC ATG GA 3′	
SCX	Quantitect primer Assay Hs_SCXB_2_SG	Not available	

## References

[1] Fenwick S.A., Hazleman B.L., Riley G.P. (2002). The vasculature and its role in the damaged and healing tendon. Arthritis Res..

[2] Pufe T., Petersen W.J., Mentlein R., Tillmann B.N. (2005). The role of vasculature and angiogenesis for the pathogenesis of degenerative tendons disease. Scand. J. Med. Sci. Sports.

[3] Docheva D., Muller S.A., Majewski M., Evans C.H. (2015). Biologics for tendon repair. Adv. Drug Deliv. Rev..

[4] Halpern B.C., Chaudhury S., Rodeo S.A. (2012). The role of platelet-rich plasma in inducing musculoskeletal tissue healing. HSS J..

[5] Zhou Y., Wang J.H. (2016). PRP Treatment Efficacy for Tendinopathy: A Review of Basic Science Studies. BioMed Res. Int..

[6] Schnabel L.V., Mohammed H.O., Miller B.J., McDermott W.G., Jacobson M.S., Santangelo K.S., Fortier L.A. (2007). Platelet rich plasma (PRP) enhances anabolic gene expression patterns in flexor digitorum superficialis tendons. J. Orthop. Res..

[7] Mazzocca A.D., McCarthy M.B., Chowaniec D.M., Dugdale E.M., Hansen D., Cote M.P., Bradley J.P., Romeo A.A., Arciero R.A., Beitzel K. (2012). The positive effects of different platelet-rich plasma methods on human muscle, bone, and tendon cells. Am. J. Sports Med..

[8] Wang X., Qiu Y., Triffitt J., Carr A., Xia Z., Sabokbar A. (2012). Proliferation and differentiation of human tenocytes in response to platelet rich plasma: An in vitro and in vivo study. J. Orthop. Res..

[9] Majewski M., Ochsner P.E., Liu F., Fluckiger R., Evans C.H. (2009). Accelerated healing of the rat Achilles tendon in response to autologous conditioned serum. Am. J. Sports Med..

[10] Dallaudiere B., Lempicki M., Pesquer L., Louedec L., Preux P.M., Meyer P., Hummel V., Larbi A., Deschamps L., Journe C. (2013). Efficacy of intra-tendinous injection of platelet-rich plasma in treating tendinosis: Comprehensive assessment of a rat model. Eur. Radiol..

[11] Spang J.T., Tischer T., Salzmann G.M., Winkler T., Burgkart R., Wexel G., Imhoff A.B. (2011). Platelet concentrate vs. saline in a rat patellar tendon healing model. Knee Surg. Sports Traumatol. Arthrosc..

[12] Lyras D.N., Kazakos K., Verettas D., Polychronidis A., Tryfonidis M., Botaitis S., Agrogiannis G., Simopoulos C., Kokka A., Patsouris E. (2009). The influence of platelet-rich plasma on angiogenesis during the early phase of tendon healing. Foot Ankle Int..

[13] Molloy T., Wang Y., Murrell G. (2003). The roles of growth factors in tendon and ligament healing. Sports Med..

[14] Andia I., Maffulli N. (2015). Muscle and tendon injuries: The role of biological interventions to promote and assist healing and recovery. Arthroscopy.

[15] Maffulli N., Del Buono A. (2012). Platelet plasma rich products in musculoskeletal medicine: Any evidence?. Surgeon.

[16] Peerbooms J.C., Sluimer J., Bruijn D.J., Gosens T. (2010). Positive effect of an autologous platelet concentrate in lateral epicondylitis in a double-blind randomized controlled trial: Platelet-rich plasma versus corticosteroid injection with a 1-year follow-up. Am. J. Sports Med..

[17] Gosens T., Peerbooms J.C., van Laar W., den Oudsten B.L. (2011). Ongoing positive effect of platelet-rich plasma versus corticosteroid injection in lateral epicondylitis: A double-blind randomized controlled trial with 2-year follow-up. Am. J. Sports Med..

[18] Sampson S., Reed M., Silvers H., Meng M., Mandelbaum B. (2010). Injection of platelet-rich plasma in patients with primary and secondary knee osteoarthritis: A pilot study. Am. J. Phys. Med. Rehabil..

[19] Wang-Saegusa A., Cugat R., Ares O., Seijas R., Cusco X., Garcia-Balletbo M. (2011). Infiltration of plasma rich in growth factors for osteoarthritis of the knee short-term effects on function and quality of life. Arch. Orthop. Trauma Surg..

[20] Filardo G., Kon E., Di Matteo B., Di Martino A., Tesei G., Pelotti P., Cenacchi A., Marcacci M. (2014). Platelet-rich plasma injections for the treatment of refractory Achilles tendinopathy: Results at 4 years. Blood Transfus..

[21] Randelli P., Arrigoni P., Ragone V., Aliprandi A., Cabitza P. (2011). Platelet rich plasma in arthroscopic rotator cuff repair: A prospective RCT study, 2-year follow-up. J. Shoulder Elbow Surg..

[22] Kaux J.F., Croisier J.L., Bruyere O., De La Cruz C.R., Forthomme B., Brabant G., Lapraille S., Lonneux V., Noel D., Le Goff C. (2015). One injection of platelet-rich plasma associated to a submaximal eccentric protocol to treat chronic jumper’s knee. J. Sports Med. Phys. Fit..

[23] Foster T.E., Puskas B.L., Mandelbaum B.R., Gerhardt M.B., Rodeo S.A. (2009). Platelet-rich plasma: From basic science to clinical applications. Am. J. Sports Med..

[24] Kushida S., Kakudo N., Morimoto N., Hara T., Ogawa T., Mitsui T., Kusumoto K. (2014). Platelet and growth factor concentrations in activated platelet-rich plasma: A comparison of seven commercial separation systems. J. Artif. Organs.

[25] Castillo T.N., Pouliot M.A., Kim H.J., Dragoo J.L. (2011). Comparison of growth factor and platelet concentration from commercial platelet-rich plasma separation systems. Am. J. Sports Med..

[26] Kruger J.P., Hondke S., Endres M., Pruss A., Siclari A., Kaps C. (2012). Human platelet-rich plasma stimulates migration and chondrogenic differentiation of human subchondral progenitor cells. J. Orthop. Res..

[27] Sandri G., Bonferoni M.C., Rossi S., Ferrari F., Mori M., Cervio M., Riva F., Liakos I., Athanassiou A., Saporito F. (2015). Platelet lysate embedded scaffolds for skin regeneration. Expert Opin. Drug Deliv..

[28] Del Fante C., Perotti C., Bonferoni M.C., Rossi S., Sandri G., Ferrari F., Scudeller L., Caramella C.M. (2011). Platelet lysate mucohadesive formulation to treat oral mucositis in graft versus host disease patients: A new therapeutic approach. AAPS PharmSciTech.

[29] Pezzotta S., Del Fante C., Scudeller L., Cervio M., Antoniazzi E.R., Perotti C. (2012). Autologous platelet lysate for treatment of refractory ocular GVHD. Bone Marrow Transplant..

[30] Dellera E., Bonferoni M.C., Sandri G., Rossi S., Ferrari F., Del Fante C., Perotti C., Grisoli P., Caramella C. (2014). Development of chitosan oleate ionic micelles loaded with silver sulfadiazine to be associated with platelet lysate for application in wound healing. Eur. J. Pharm. Biopharm..

[31] El Backly R., Ulivi V., Tonachini L., Cancedda R., Descalzi F., Mastrogiacomo M. (2011). Platelet lysate induces in vitro wound healing of human keratinocytes associated with a strong proinflammatory response. Tissue Eng. Part A.

[32] Ranzato E., Mazzucco L., Patrone M., Burlando B. (2009). Platelet lysate promotes in vitro wound scratch closure of human dermal fibroblasts: Different roles of cell calcium, P38, ERK and PI3K/AKT. J. Cell. Mol. Med..

[33] Burnouf T., Strunk D., Koh M.B., Schallmoser K. (2016). Human platelet lysate: Replacing fetal bovine serum as a gold standard for human cell propagation?. Biomaterials.

[34] Schallmoser K., Bartmann C., Rohde E., Reinisch A., Kashofer K., Stadelmeyer E., Drexler C., Lanzer G., Linkesch W., Strunk D. (2007). Human platelet lysate can replace fetal bovine serum for clinical-scale expansion of functional mesenchymal stromal cells. Transfusion.

[35] Strunk D., Lozano M., Marks D.C., Loh Y.S., Gstraunthaler G., Schennach H., Rohde E., Laner-Plamberger S., Oller M., Nystedt J. (2017). International Forum on GMP-grade human platelet lysate for cell propagation: Summary. Vox Sang..

[36] Bernardi M., Agostini F., Chieregato K., Amati E., Durante C., Rassu M., Ruggeri M., Sella S., Lombardi E., Mazzucato M. (2017). The production method affects the efficacy of platelet derivatives to expand mesenchymal stromal cells in vitro. J. Transl. Med..

[37] Muraglia A., Nguyen V.T., Nardini M., Mogni M., Coviello D., Dozin B., Strada P., Baldelli I., Formica M., Cancedda R. (2017). Culture Medium Supplements Derived from Human Platelet and Plasma: Cell Commitment and Proliferation Support. Front. Bioeng. Biotechnol..

[38] Nguyen V.T., Cancedda R., Descalzi F. (2017). Platelet lysate activates quiescent cell proliferation and reprogramming in human articular cartilage: Involvement of hypoxia inducible factor 1. J. Tissue Eng. Regen. Med..

[39] Wang T.J., Chen M.S., Chou M.L., Lin H.C., Seghatchian J., Burnouf T. (2017). Comparison of three human platelet lysates used as supplements for in vitro expansion of corneal endothelium cells. Transfus. Apher. Sci..

[40] Del Bue M., Ricco S., Conti V., Merli E., Ramoni R., Grolli S. (2007). Platelet lysate promotes in vitro proliferation of equine mesenchymal stem cells and tenocytes. Vet. Res. Commun..

[41] Sellberg F., Berglund E., Ronaghi M., Strandberg G., Lof H., Sommar P., Lubenow N., Knutson F., Berglund D. (2016). Composition of growth factors and cytokines in lysates obtained from fresh versus stored pathogen-inactivated platelet units. Transfus. Apher. Sci..

[42] Kruger J.P., Freymannx U., Vetterlein S., Neumann K., Endres M., Kaps C. (2013). Bioactive factors in platelet-rich plasma obtained by apheresis. Transfus. Med. Hemother..

[43] Liu X., Yang Y., Niu X., Lin Q., Zhao B., Wang Y., Zhu L. (2017). An in situ photocrosslinkable platelet rich plasma—Complexed hydrogel glue with growth factor controlled release ability to promote cartilage defect repair. Acta Biomater..

[44] Kobayashi E., Fluckiger L., Fujioka-Kobayashi M., Sawada K., Sculean A., Schaller B., Miron R.J. (2016). Comparative release of growth factors from PRP, PRF, and advanced-PRF. Clin. Oral Investig..

[45] Kobayashi E., Fujioka-Kobayashi M., Sculean A., Chappuis V., Buser D., Schaller B., Dori F., Miron R.J. (2017). Effects of platelet rich plasma (PRP) on human gingival fibroblast, osteoblast and periodontal ligament cell behaviour. BMC Oral Health.

[46] Sundman E.A., Cole B.J., Fortier L.A. (2011). Growth factor and catabolic cytokine concentrations are influenced by the cellular composition of platelet-rich plasma. Am. J. Sports Med..

[47] Magalon J., Bausset O., Serratrice N., Giraudo L., Aboudou H., Veran J., Magalon G., Dignat-Georges F., Sabatier F. (2014). Characterization and comparison of 5 platelet-rich plasma preparations in a single-donor model. Arthroscopy.

[48] Giusti I., D’Ascenzo S., Manco A., Di Stefano G., Di Francesco M., Rughetti A., Dal Mas A., Properzi G., Calvisi V., Dolo V. (2014). Platelet concentration in platelet-rich plasma affects tenocyte behavior in vitro. BioMed Res. Int..

[49] Sadoghi P., Lohberger B., Aigner B., Kaltenegger H., Friesenbichler J., Wolf M., Sununu T., Leithner A., Vavken P. (2013). Effect of platelet-rich plasma on the biologic activity of the human rotator-cuff fibroblasts: A controlled in vitro study. J. Orthop. Res..

[50] Arslan E., Nellesen T., Bayer A., Prescher A., Lippross S., Nebelung S., Jahr H., Jaeger C., Huebner W.D., Fischer H. (2016). Effect of platelet mediator concentrate (PMC) on Achilles tenocytes: An in vitro study. BMC Musculoskelet. Disord..

[51] Jo C.H., Kim J.E., Yoon K.S., Shin S. (2012). Platelet-rich plasma stimulates cell proliferation and enhances matrix gene expression and synthesis in tenocytes from human rotator cuff tendons with degenerative tears. Am. J. Sports Med..

[52] De Mos M., van der Windt A.E., Jahr H., van Schie H.T., Weinans H., Verhaar J.A., van Osch G.J. (2008). Can platelet-rich plasma enhance tendon repair? A cell culture study. Am. J. Sports Med..

[53] Maffulli N., Ewen S.W., Waterston S.W., Reaper J., Barrass V. (2000). Tenocytes from ruptured and tendinopathic achilles tendons produce greater quantities of type III collagen than tenocytes from normal achilles tendons. An in vitro model of human tendon healing. Am. J. Sports Med..

[54] Schweitzer R., Chyung J.H., Murtaugh L.C., Brent A.E., Rosen V., Olson E.N., Lassar A., Tabin C.J. (2001). Analysis of the tendon cell fate using Scleraxis, a specific marker for tendons and ligaments. Development.

[55] Alberton P., Popov C., Pragert M., Kohler J., Shukunami C., Schieker M., Docheva D. (2012). Conversion of human bone marrow-derived mesenchymal stem cells into tendon progenitor cells by ectopic expression of scleraxis. Stem Cells Dev..

[56] Murchison N.D., Price B.A., Conner D.A., Keene D.R., Olson E.N., Tabin C.J., Schweitzer R. (2007). Regulation of tendon differentiation by scleraxis distinguishes force-transmitting tendons from muscle-anchoring tendons. Development.

[57] Sharma P., Maffulli N. (2006). Biology of tendon injury: Healing, modeling and remodeling. J. Musculoskelet. Neuronal Interact..

[58] Moojen D.J., Everts P.A., Schure R.M., Overdevest E.P., van Zundert A., Knape J.T., Castelein R.M., Creemers L.B., Dhert W.J. (2008). Antimicrobial activity of platelet-leukocyte gel against Staphylococcus aureus. J. Orthop. Res..

[59] Borregaard N., Cowland J.B. (1997). Granules of the human neutrophilic polymorphonuclear leukocyte. Blood.

[60] John T., Lodka D., Kohl B., Ertel W., Jammrath J., Conrad C., Stoll C., Busch C., Schulze-Tanzil G. (2010). Effect of pro-inflammatory and immunoregulatory cytokines on human tenocytes. J. Orthop. Res..

[61] Cai Y.Z., Zhang C., Lin X.J. (2015). Efficacy of platelet-rich plasma in arthroscopic repair of full-thickness rotator cuff tears: A meta-analysis. J. Shoulder Elbow Surg..

[62] Li X., Xu C.P., Hou Y.L., Song J.Q., Cui Z., Yu B. (2014). Are platelet concentrates an ideal biomaterial for arthroscopic rotator cuff repair? A meta-analysis of randomized controlled trials. Arthroscopy.

[63] Saltzman B.M., Jain A., Campbell K.A., Mascarenhas R., Romeo A.A., Verma N.N., Cole B.J. (2016). Does the Use of Platelet-Rich Plasma at the Time of Surgery Improve Clinical Outcomes in Arthroscopic Rotator Cuff Repair When Compared With Control Cohorts? A Systematic Review of Meta-analyses. Arthroscopy.

[64] Warth R.J., Dornan G.J., James E.W., Horan M.P., Millett P.J. (2015). Clinical and structural outcomes after arthroscopic repair of full-thickness rotator cuff tears with and without platelet-rich product supplementation: A meta-analysis and meta-regression. Arthroscopy.

[65] Flury M., Rickenbacher D., Schwyzer H.K., Jung C., Schneider M.M., Stahnke K., Goldhahn J., Audige L. (2016). Does Pure Platelet-Rich Plasma Affect Postoperative Clinical Outcomes After Arthroscopic Rotator Cuff Repair? A Randomized Controlled Trial. Am. J. Sports Med..

[66] Zhang J., Middleton K.K., Fu F.H., Im H.J., Wang J.H. (2013). HGF mediates the anti-inflammatory effects of PRP on injured tendons. PLoS ONE.

[67] Klatte-Schulz F., Pauly S., Scheibel M., Greiner S., Gerhardt C., Schmidmaier G., Wildemann B. (2012). Influence of age on the cell biological characteristics and the stimulation potential of male human tenocyte-like cells. Eur. Cells Mater..

[68] Xiong G., Lingampalli N., Koltsov J.C.B., Leung L.L., Bhutani N., Robinson W.H., Chu C.R. (2017). Men and Women Differ in the Biochemical Composition of Platelet-Rich Plasma. Am. J. Sports Med..

[69] Fekete N., Gadelorge M., Furst D., Maurer C., Dausend J., Fleury-Cappellesso S., Mailander V., Lotfi R., Ignatius A., Sensebe L. (2012). Platelet lysate from whole blood-derived pooled platelet concentrates and apheresis-derived platelet concentrates for the isolation and expansion of human bone marrow mesenchymal stromal cells: Production process, content and identification of active components. Cytotherapy.

[70] Pauly S., Klatte F., Strobel C., Schmidmaier G., Greiner S., Scheibel M., Wildemann B. (2010). Characterization of tendon cell cultures of the human rotator cuff. Eur. Cells Mater..

